# Electrolyte disorders with platinum-based chemotherapy: mechanisms, manifestations and management

**DOI:** 10.1007/s00280-017-3392-8

**Published:** 2017-07-20

**Authors:** Bryan Oronsky, Scott Caroen, Arnold Oronsky, Vaughn E. Dobalian, Neil Oronsky, Michelle Lybeck, Tony R. Reid, Corey A. Carter

**Affiliations:** 1 EpicentRx Inc, 4445 Eastgate Mall, Suite 200, San Diego, CA 92121 USA; 2 InterWest Partners, 2710 Sand Hill Road #200, Menlo Park, CA 94025 USA; 3 Beaches Family Medicine, 465 3rd St N, Jacksonville Beach, FL 32250 USA; 4 Walter Reed National Military Medical Center, 8901 Wisconsin Ave, Bethesda, MD 20889 USA; 5 Moores Cancer Center, University of California San Diego, 3855 Health Sciences Dr, La Jolla, CA 92093 USA; 6 CFLS Data, 800 W El Camino Real, Suite 180, Mountain View, CA 94040 USA

**Keywords:** Electrolyte, Platinum, Chemotherapy, Cisplatin, Toxicity

## Abstract

Platinum chemotherapy, particularly cisplatin, is commonly associated with electrolyte imbalances, including hypomagnesemia, hypokalemia, hypophosphatemia, hypocalcemia and hyponatremia. The corpus of literature on these dyselectrolytemias is large; the objective of this review is to synthesize the literature and summarize the mechanisms responsible for these particular electrolyte disturbances in the context of platinum-based treatment as well as to present the clinical manifestations and current management strategies for oncologists and primary care physicians, since the latter are increasingly called on to provide care for cancer patients with medical comorbidities. Correct diagnosis and effective treatment are essential to improved patient outcomes.

## Introduction

Platinum-based chemotherapy with cisplatin (the prototype of platinum agents) and its derivatives, carboplatin and oxaliplatin, is traditionally the first-line cytotoxic treatment of lung, colorectal, ovarian, breast, head/neck, bladder and testicular cancers [1].

An expected side effect of these widely administered platinum-containing regimens is electrolyte disturbances, in particular low magnesium with cisplatin [2]. However, since the signs and symptoms of dyselectrolytemias may be subtle and nonspecific, overlapping with and, therefore, potentially attributed to other comorbid conditions, including the cancer itself, a high index of suspicion is required to make the diagnosis. Besides the potential to adversely affect quality of life, misdiagnosis or delay may also lead to therapy dose reductions, discontinuations or even death, which makes prompt recognition and correction important.

Given the projected shortfall in the workforce of oncologists [3], the responsibility to recognize and manage the comorbidities of cancer patients may increasingly fall to family practitioners and general internists; hence, this review of the mechanisms, manifestations and management strategies for each of the platinum-induced electrolyte disturbances (magnesium, sodium, potassium, phosphate and calcium) is intended for a wider audience of clinicians, especially since a comparably general overview was not available/not found on a PubMed literature search. In accordance with its relative importance, this review begins with a discussion of low magnesium levels.

## Low magnesium

### Background

The fourth most abundant cation, and crucial cofactor in >300 enzymatic reactions including the sodium–potassium pump or Na–K ATPase [4], magnesium is predominantly located intracellularly or deposited in bone; thus, serum levels are poor indicators of total body stores and the presence of hypomagnesemia defined as a concentration <1.5 mEq/L usually indicates a more severe underlying cellular deficit [4]. Conversely, tissue deficiency may be present despite normal serum levels.

Therefore, hypomagnesemia and magnesium deficiency are not interchangeable or synonymous terms even though they are commonly used as such. Plasma concentrations are reported in mEq/L versus units or grams for replacement therapy. Only 55% of the total serum concentration circulates in the ionized or physiologically active form, with the rest bound to albumin or chelated to anions (e.g., phosphate, oxalate, citrate or sulfate [5]), and unlike techniques for measuring ionized calcium, ionized magnesium testing is not routinely available [6]. Because approximately 30% of magnesium is bound to albumin, hypoalbuminemic states may lead to low magnesium values [7].

**Fig. 1:**
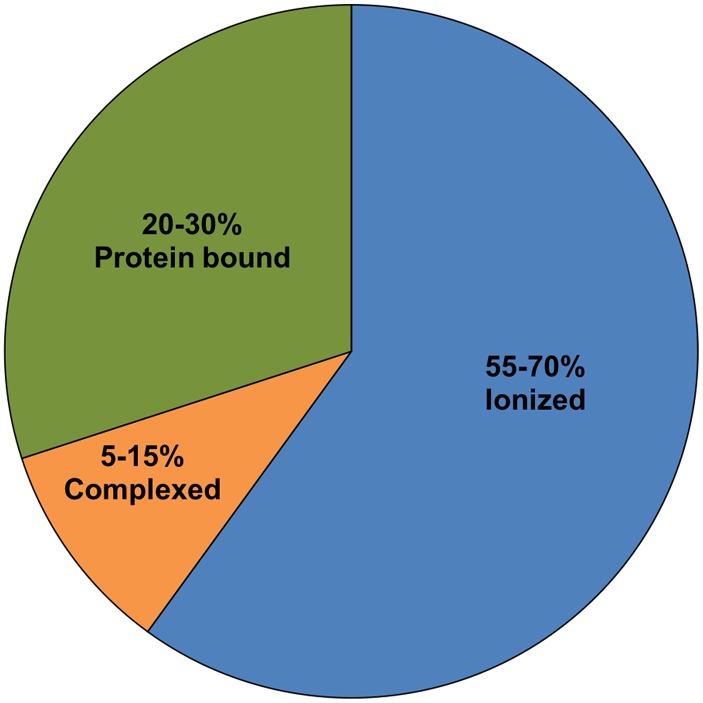
Total serum magnesium presented in three different states. Because of different measurement methods, results published for each state of serum magnesium vary considerably. Therefore, a range for every state is provided

Total serum magnesium is present in three different states (Fig. 1). Because of different measurement methods, results published for each state of serum magnesium vary considerably. Therefore, a range for every state is provided.

Magnesium deficiency is fairly widespread even in the absence of platinum therapy [8]; however, it is usually not recognized and therefore not found or corrected. Gastrointestinal and renal losses are the two major mechanisms responsible for the induction of magnesium deficiency and hypomagnesemia [9]. Because the mobilization of magnesium from the skeleton is exceedingly slow [10], magnesium deficiency not only occurs relatively quickly with minimal losses but also takes longer to normalize.

The concentration of magnesium (Mg^++^) significantly influences serum levels of other electrolytes such as potassium, calcium and phosphate [11], which points out the centrality of its position and role. In terms of this review, a low magnesium state is particularly associated with cisplatin (and to a much lesser extent carboplatin [12]) therapy, more so than any other electrolyte deficiency, affecting 40–90% of patients [13] as opposed to 10% of patients treated with carboplatin [14].

### Mechanism

Platinum-induced hypomagnesemia, which has been reported to persist for up to 6 years after cessation of treatment [15], is primarily attributed to renal magnesium wasting and/or reduced intestinal absorption. Additional factors discussed below that can exacerbate magnesium deficiency include drugs, endocrine causes and alcoholism (Table 1).

**Table 1:** Causes of hypomagnesemia

Redistribution of magnesium
Refeeding and insulin therapy
Hungry bone syndrome
Correction of acidosis
Catecholamine excess
Massive blood transfusion
Gastrointestinal causes
Reduced intake
Mg-free intravenous fluids
Dietary deficiency
Low oxalate diet
Cellulose phosphate
Reduced absorption
Malabsorption syndrome
Chronic diarrhea
Intestinal resection
Primary infantile hypomagnesemia
Renal loss
Reduced sodium reabsorption
Saline infusion
Diuretics
Renal disease
Post-obstructive nephropathy
Post-renal transplantation
Dialysis
Diuretic phase of acute renal failure
Inherited disorders
Bartter’s syndrome
Gitelman’s syndrome
Endocrine causes
Hypercalcaemia
Primary hyperparathyroidism
Malignant hypercalcaemia
Hyperthyroidism
Hyperaldosteronism
Diabetes mellitus
Alcoholism
Drugs
Diuretics
Cytotoxic drugs: cisplatin, carboplatin, gallium nitrate
Antimicrobial agents
Aminoglycosides: gentamicin, tobramycin, amikacin
Antituberculous drugs: viomycin, capreomycin
Immunosuppressants: cyclosporin, ritodrine
Beta-adrenergic agonists: theophylline, salbutamol, riniterol
Other drugs
Amphotericin B
Pentamidine
Foscarnet
Pamidronate
Anascrine

**Fig. 2:**
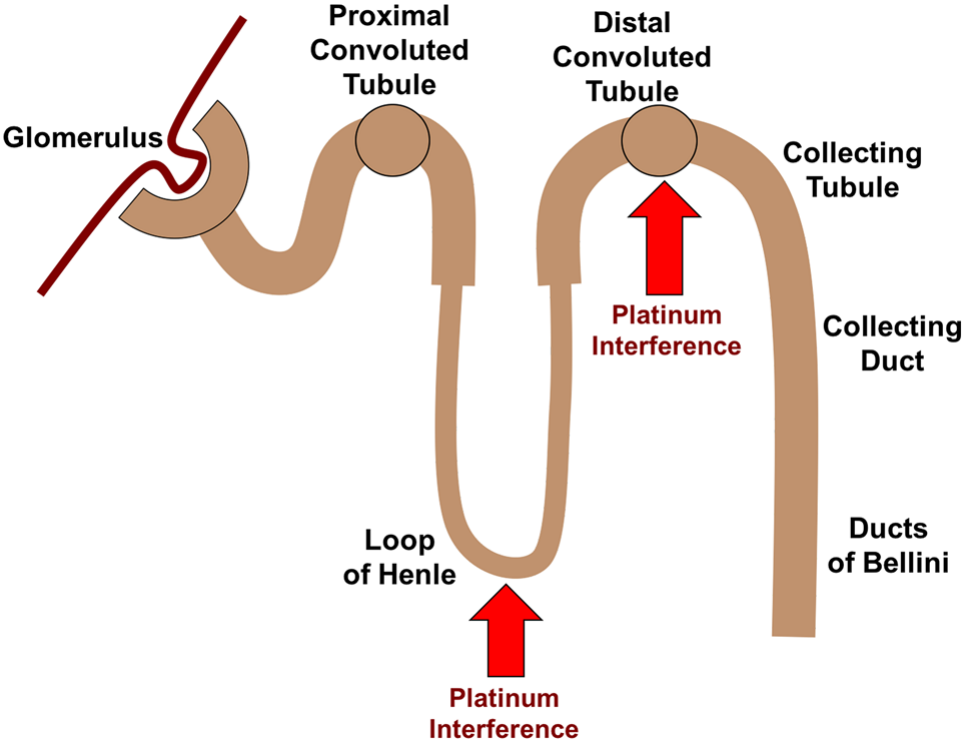
Nephron structure and sites of platinum interference with magnesium absorption

- Renal wasting: Unlike oxaliplatin or carboplatin that are predominately bound to plasma proteins, cisplatin is largely unbound [16] and freely filtered through the glomerulus, where it subsequently accumulates in kidney tubular cells through organic cation transporters (OCT [17]) and potentially mediates nephrotoxicity. Direct injury to magnesium reabsorption in the ascending limb of loop of Henle, as well as the distal tubule, is the possible mechanisms by which cisplatin specifically induces hypomagnesemia [18] (Fig. 2).
- GI losses: In addition to renal tubular damage, platinum-induced magnesium loss may also take place from the gut. The Mg concentration of upper and lower intestinal tract fluids is approximately 0.5 mmol/L and 15 mEq/L [19], respectively, and, therefore, since vomiting, diarrhea and anorexia are common occurrences with platinum therapy so is magnesium depletion.
- Other

## Medications

In addition to platinum agents, a variety of medications including loop and thiazide diuretics, antibiotics, bisphosphonates, corticosteroids and beta-2 agonists may cause magnesium wasting, which has implications for cardiologists, pulmonologists, internists, hospitalists and family practitioners that may be providing ancillary care to these patients. A more complete list of medications and general causes of hypomagnesemia is provided in Table 1.

### Loop and thiazide diuretics

Loop diuretics (e.g., furosemide) cause magnesium depletion via inhibition of magnesium transport in the ascending loop of Henle, especially during long-term use [20], while thiazide diuretics (e.g., HCTZ) lead to urinary magnesium losses [21] through increased solute diuresis. By the same mechanism, polyuric states due to diabetes mellitus (osmotic diuresis), post-obstructive diuresis, mannitol administration, hyperaldosteronism and volume expansion from normal saline, a common cause of hypomagnesemia in hospitalized patients, may induce magnesium deficiency. If possible, loop or thiazide diuretics should be discontinued during platinum therapy and replaced with potassium-sparing diuretics such as spironolactone, triamterene or amiloride [22], which also exert magnesium-sparing properties.

### Aminoglycosides

These antibiotics including gentamicin, tobramycin and amikacin, which accumulate in and preferentially damage proximal tubular cells, are known to cause hypomagnesemia, and the use of alternative agents should be considered, if possible, during platinum therapy [23].

### Bisphosphonates

Hypomagnesemia may occur with bisphosphonates as a result of binding of the bisphosphonates to magnesium cations [24].

### β_2_-Agonists and corticosteroids

Patients with lung cancer and COPD under long term or acute therapy with inhaled or systemic corticosteroids and β_2_-agonists are also at risk for hypomagnesemia. Corticosteroids (e.g., dexamethasone) may also be given as a premedication to prevent cisplatin-induced nausea and vomiting. Close monitoring and regular supplementation with magnesium should be considered in these cases: beta-adrenergic stimulation not only induces an extracellular to intracellular shift of magnesium ions but also results in higher levels of free fatty acids that chelate Mg^+2^; [25, 26] corticosteroid use, which also leads to bone loss from renal excretion and suppressed intestinal absorption of calcium, may secondarily cause a net negative magnesium balance due to increased bone resorption and renal wasting.

### Insulin therapy

Insulin modulates the shift of magnesium from extracellular to intracellular space, which may exacerbate low Mg^++^ levels [27]. In addition, poorly controlled diabetes may result in renal magnesium wasting secondary to the osmotic diuresis from hyperglycosuria. Evaluation of magnesium status in diabetic patients, especially poorly controlled and/or insulin-dependent diabetic patients, is recommended.

### Anti-EGFR treatment

Cetuximab (Erbitux) and panitumumab (Vectibix), monoclonal antibodies, which bind specifically to EGFR, are associated with a high rate of hypomagnesemia (60% in the case of cetuximab). The mechanism is related to inhibition of the Mg^2+^ channel TRPM6 (Transient Receptor Potential Melastatin subtype 6/7), which regulates magnesium absorption in the gut and renal tubules [28]. Suspicion of Mg^2+^ deficiency should be particularly elevated in patients with recurrent/metastatic head and neck squamous cell carcinoma (HNSCC [29]) where cetuximab in combination with platinum and 5FU is administered as the first-line standard of care.

### Proton pump inhibitors (PPIs)

As discussed above, magnesium absorption occurs in the gut primarily through the Mg2 + influx channel, Transient Receptor Potential Melastatin 6 (TRPM6/7) [30], also found in the distal renal tubule, which is pH sensitive. PPI use, which leads to alkalization of the intestinal lumen, changes the TRPM6/7 channel affinity for Mg^2+^ and inhibits magnesium absorption [31]. In this way, PPIs such as lansoprazole, omeprazole, rabeprazole and esomeprazole may induce hypomagnesemia refractory to oral supplementation [32].

## Alcoholism

Hypomagnesemia and inappropriate magnesiuria have been described in alcoholic patients possibly due to a direct magnesiuric effect of alcohol consumption or decreased nutritional intake [33]. Therefore, platinum-treated cancer patients should be screened for alcohol consumption.

## Hypercalcemia

Hypercalcemia, which affects up to 10 to 30% of cancer patients primarily due to increased osteoclastic bone resorption, induces hypomagnesemia because calcium and magnesium compete for transport in the thick ascending loop of Henle [34]. The mainstay of treatment is intravenous (IV) bisphosphonates, which may themselves lower magnesium levels.

## Blood transfusion

Hypomagnesemia may develop with blood transfusions due to the presence of citrate, which chelates magnesium (and calcium) cations.

### Manifestations

Early signs of magnesium deficiency including loss of appetite, nausea, vomiting, fatigue and weakness [35] are confounding because these same adverse events commonly occur with platinum therapy. As magnesium deficiency worsens a depolarization shift of the membrane occurs, potentially resulting in behavioral changes (e.g., confusion, agitation, depression), neuromuscular excitability (e.g., numbness, tingling, hyperactive deep tendon reflexes, muscle contractions, cramps and seizures) and cardiac effects including arrhythmias [36] (ventricular tachycardia, Torsade de pointes, ventricular fibrillation), abnormal electrical activity (prolonged QT and QU interval), potentiation of digitalis effects and vascular tone [37]. With severe hypomagnesemia, low levels of calcium and potassium in the blood may be observed and, on physical exam, similar to manifestations of hypocalcemia, the Chvostek (twitching of facial muscles in response to tapping over the facial nerve in front of the ear) and Trousseau (spasm of the hands induced by an inflated blood pressure cuff on the arm) signs of latent tetany may be elicited [38].

However, it is important to note that even in severely hypomagnesemic patients, clinical signs or red flags may be few, non-obvious or absent [39]. In addition, the presence of symptoms is more common in patients that experience a rapid rather than a gradual decrease in serum magnesium. Due to the lack of accurate diagnostic studies, a presumptive diagnosis of low magnesium is often warranted whether or not symptoms are present since, if left untreated, the potential exists for catastrophic outcomes (e.g., death from cardiac arrhythmias).

### Management

Traditionally, the management of hypomagnesemia has been based on the NCI grading criteria shown below [40]:

- Grade 0 = Within normal limits (1.8–2.1 mg/dL).
- Grade 1 = 1.2 mg/dL to 1.8 mg/dL.
- Grade 2 = 0.9–.2 mg/dL.
- Grade 3 = 0.7–0.9 mg/dL.
- Grade 4 = Less than 0.7 mg/dL.
- Grade 5 = Death.

However, since measurement of serum levels may not be accurate or diagnostic, the decision to treat should be based on the strength of belief and clinical judgment (in other words, pretest probability) taking into account comorbidities such as diabetes or heart disease, which make dangerous arrhythmias more likely, concomitant medications that predispose to magnesium losses such as diuretics, bisphosphonates, proton pump inhibitors, beta-2 agonists and corticosteroids, and miscellaneous factors including alcohol intake, blood transfusions and calcium levels. Electrolyte abnormalities that converge on hypomagnesemia and serve as important diagnostic clues to its presence include concomitant hypokalemia (due to impaired Na–K–ATPase and urinary potassium wasting) and hypocalcemia (due both to lower parathyroid hormone secretion and end-organ resistance to its effect [41]); therefore, measurement of potassium and calcium levels should be sought as supportive evidence.

The two preferred agents for repletion are i.v. magnesium sulfate (2–4 g) and oral magnesium oxide [42]. Additional oral preparations include magnesium gluconate and magnesium sulfate along with sustained-release preparations such as Slow-Mag and Mag-Tab SR. The usual therapeutic dose of oral magnesium oxide is 400–800 mg per day with the caveat that at doses >400 mg diarrhea is likely to result, which increases magnesium excretion. Similar to glucocorticoid supplementation therapy during surgery or medical illness, patients may require additional magnesium doses during acute ill health, especially when vomiting and diarrhea are present.

Because excess magnesium is efficiently eliminated in the urine and plasma levels are closely maintained between 1.5 and 2.1 mEq/L, hypermagnesemia (>2.6 mg/dL) is a rare complication except in the setting of renal insufficiency (high creatinine or low glomerular filtration rate), which is the most common acute toxicity of platinum agents. Initial symptoms of hypermagnesemia, such as nausea, vomiting, lethargy and weakness are nonspecific but may rapidly progress to respiratory depression, prolonged PR and QT intervals on an ECG, hypotension, ventricular arrhythmias, cardiac arrest and death [43]. When renal function is impaired, treatment includes discontinuation of magnesium administration, increased fluid intake and loop diuretics. Calcium gluconate, a magnesium antagonist, is generally reserved for life-threatening symptoms, such as arrhythmia or severe respiratory depression.

In addition to low magnesium, platinum agents may also cause other electrolyte abnormalities including hypokalemia, hypocalcemia, hypophosphatemia and hyponatremia, discussed below. Hypokalemia, hypocalcemia and hypophosphatemia are considered together because of their relationship to magnesium deficiency and refractoriness to correction prior to magnesium repletion. However, even in the absence of low magnesium, renal potassium, calcium and phosphate wasting may occur as a result of platinum-mediated damage to tubular membranes.

## Hypokalemia, hypocalcemia and hypophosphatemia

### Background and mechanism

#### K^+^

The major intracellular cation, responsible for the maintenance of a normal charge difference between intracellular and extracellular environments [44], which, in turn, influences the excitability of muscle and nerve tissue as well as pH, only 2% of potassium is extracellular. Normal serum potassium is strictly controlled within a narrow range 3.5–5.2 mmol/L. Homeostasis is maintained by renal and intestinal excretion as well as shifts between the extracellular and intracellular fluid compartments. Since platinum agents are nephrotoxic, emetogenic and diarrheagenic, they have the potential to induce potassium wasting [45].

Low potassium tends to coexist with low magnesium since magnesium is a cofactor of ATP, with the Mg^2+^ ion bound to the negatively charged oxygen atoms of the phosphate groups [46]. Therefore, when the concentration of ionized magnesium is decreased due to platinum administration, the Na^+^–K–ATPase [47] (i.e., sodium potassium pump) is inhibited; as a result, the cells lose potassium, which is excreted in the urine due to release of the Mg^2+^-dependent inhibition of the potassium channels of the ascending limb cells [48]. Hence, the hypokalemia, which is relatively refractory to potassium supplementation, requires correction of the magnesium deficiency [49]. A partial list of causes, which may precipitate or exacerbate hypokalemia, are shown in Table 2.

**Table 2:** Hypokalemia causes (adapted from http://www.clevelandclinicmeded.com/medicalpubs/diseasemanagement/nephrology/hypokalemia-and-hyperkalemia/)

Increased excretion
GI losses
Diarrhea, laxative abuse, gastric suctioning
Renal losses
Loop and thiazide diuretics
Osmotic diuresis (uncontrolled diabetes)
Post-obstruction
Hyperaldosteronism
Primary hyperaldosteronism
Corticosteroids
Magnesium depletion
Platinum agents
Alcoholism
Cellular shifts
Insulin administration
β-Adrenergic agonists (bronchodilators, decongestants, theophylline)
Acute catecholamine surge (e.g., myocardial infarction)

### Manifestations

Mild hypokalemia >2.5 mmol/L is often symptomatic [50]. Below 2.5 mmol/L, the leading symptoms of hypokalemia are muscle weakness, muscle pain, ileus and cramps [51]. With severe hypokalemia, rhabdomyolysis or paralysis may occur.

### Workup and management

#### K^+^

**Fig. 3:**
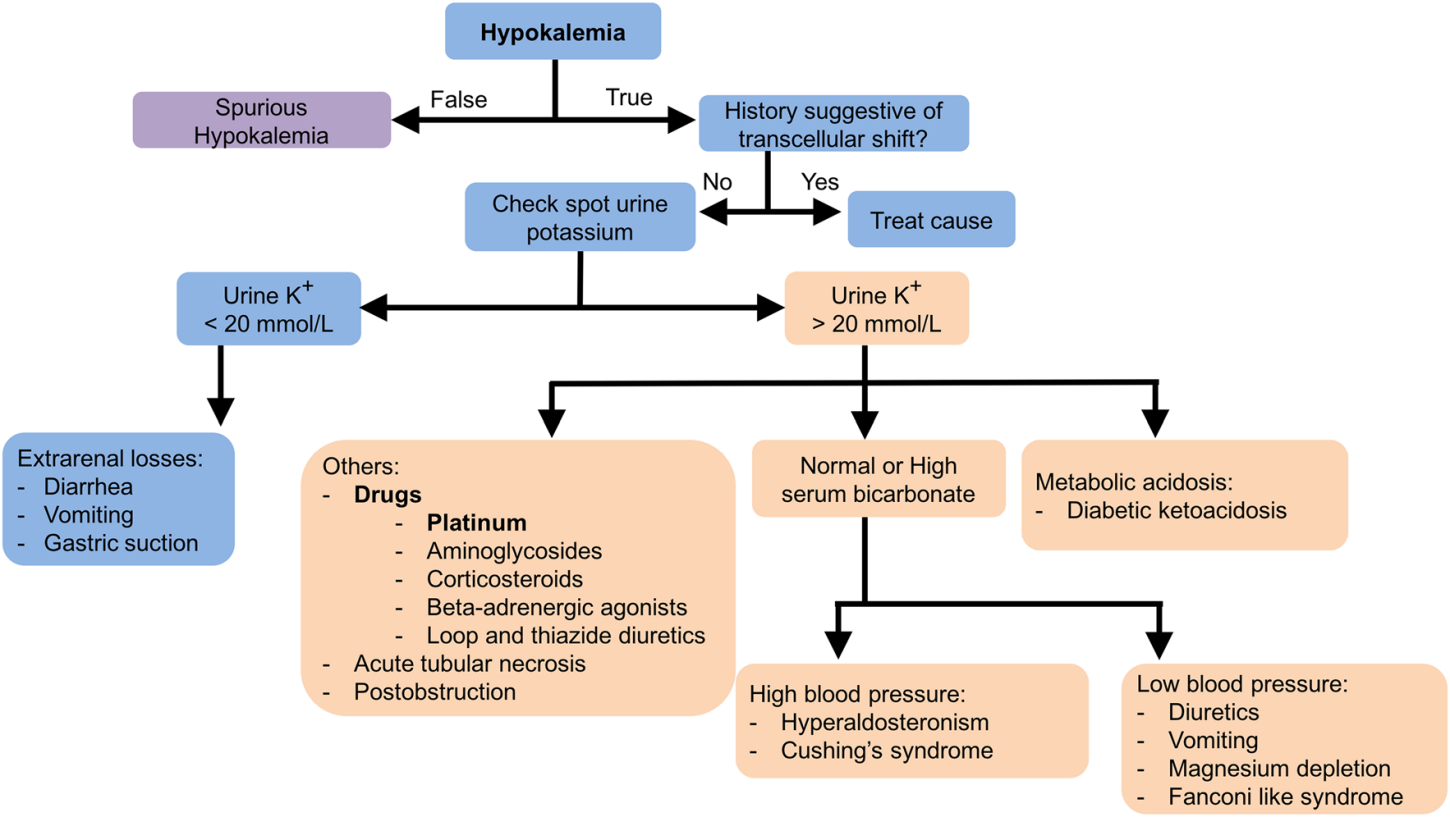
Hypokalemia diagnostic algorithm

When hypokalemia is discovered, a careful history and physical examination is warranted (see Fig. 3) to look for potential contributory factors such as vomiting, diarrhea and hypertension (due to paraneoplastic hyperaldosteronism or Cushing’s Syndrome) provided prompt replacement is not required. Factitious or spurious hypokalemia due to laboratory error or severe leukocytosis should be ruled out. If it is ruled out, the evaluation should include measurement of spot urine K^+^ and, potentially, a plasma aldosterone and ACTH.

Since low magnesium leads to renal potassium wasting and refractoriness to replacement, serum magnesium levels should be checked in all cases and replaced if warranted. Loop or thiazide diuretics should be discontinued if possible or combined with potassium-sparing amiloride, triamterene or spirinolactone. In emergent cases, which are rare, such as hypokalemic paralysis or cardiac arrhythmias, IV replacement with KCl is indicated [52]. Otherwise, oral KCl is preferred except if metabolic acidosis is present in which case potassium bicarbonate or potassium citrate is used.

#### Ca^+2^

Calcium is a divalent cation, with 99% stored in the bones and teeth. The extracellular fraction, which is tightly regulated within narrow limits (normal serum calcium concentrations range from 8.5 to 10.5 mg/dL) via parathyroid hormone (PTH) and vitamin D, includes three forms: ionized (50%), protein-bound (40%) and complexed (10%). Because only diffusible calcium (ionized plus complexed) can cross cell membranes [53], which, in turn, influences PTH secretion, the measurement of ionized rather than total calcium has greater clinical relevance.

In platinum-treated patients, hypocalcemia is principally related to hypomagnesemia (although severe hypermagnesemia may have the same effect) since magnesium is a required cofactor for parathyroid hormone (PTH) release. PTH, the principal regulator of calcium homeostasis, mobilizes calcium from bone, stimulates the renal distal tubule reabsorption of calcium and increases the synthesis of 1,25-dihydroxyvitamin-D3 for intestinal absorption of calcium and phosphate [54]. Thus, hypocalcemia is resistant to treatment until magnesium concentrations have been returned to normal [55].

In addition to decreased PTH secretion, which may be related to hypoparathyroidism, due to the removal of the parathyroid glands during surgery for the thyroid or the parathyroid, hypocalcemia may be caused by chelation with phosphates, anticoagulants (e.g., citrate, EDTA) and free fatty acids from acute pancreatitis as well as extensive osteoblastic metastases and sepsis. A partial list of causes, which may precipitate or exacerbate hypocalcemia, are shown in Table 3.

**Table 3:** Hypocalcemia causes (adapted from http://www.clevelandclinicmeded.com/medicalpubs/diseasemanagement/endocrinology/hypocalcemia/)

Decreased entry	Loss	Miscellaneous
Hypoparathyroidism	Chelation	Sepsis
	Hyperphosphatemia (e.g., from tumor Lysis syndrome or rhabdomyolysis)	
	Anticoagulants (e.g., citrate, EDTA)	
	Pancreatitis	
	Fluoride	
	Oxaliplatin	
Magnesium depletion	Increased urinary excretion	
	Platinum agents	
	5-FU	
Vitamin D deficiency (e.g., sunlight deprivation)	Osteoblastic metastases	
Severe hypermagnesemia	Alcoholism	
Diarrhea		

As the standard first-line chemotherapy for the treatment of colorectal cancer, which is associated with intractable peripheral neuropathy [56], oxaliplatin deserves special mention as a cause of hypocalcemia because it is metabolized to oxalate, which chelates calcium and magnesium. Since hypocalcemia has been associated with peripheral neuropathy, a common practice on the day of oxaliplatin dosing is to infuse calcium and magnesium, even though a recent clinical trial, which randomized patients to receive intravenous CaMg (1 g calcium gluconate, 1 g magnesium sulfate) or placebo before and after oxaliplatin, showed no benefit [57].

### Manifestations

Mild hypocalcemia is usually asymptomatic, but perioral numbness and carpopedal spasms of the hands and feet, which in some cases may progress to tetany, are typical of large or abrupt changes in ionized calcium [58]. Chvostek’s sign (facial twitch from tapping on the facial nerve near the temporal mandibular joint) and Trousseau’s sign (hand flexion after inflating a blood pressure cuff on the arm for 3 min) are both clinical tests for this increased neuromuscular reactivity. Hypocalcemia may also progress to heart block and ventricular fibrillation.

### Workup and management

**Fig. 4:**
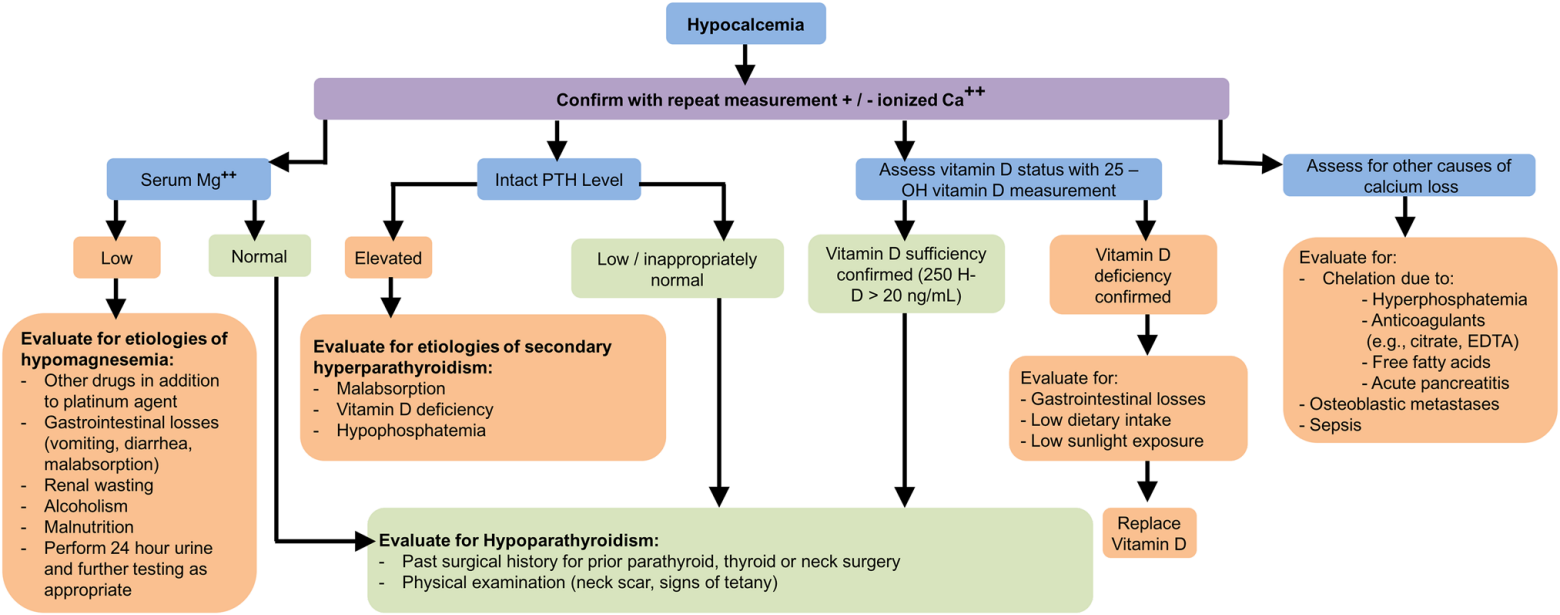
Hypocalcemia diagnostic algorithm

The workup for hypocalcemia should include measurement of ionized calcium, serum magnesium, intact PTH (iPTH) and vitamin D (Fig. 4).

The correction of magnesium and phosphate should precede the correction of calcium. 1–2 g of elemental calcium per day may be sufficient for mild hypocalcemia. Intravenous calcium infusions are only indicated in the setting of symptomatic or severe (<7.0 mg/dL or <0.8 mmol/L) hypocalcemia. The preferred form is calcium gluconate. If appropriate, thiazide diuretics, which diminish the renal excretion of calcium, should be used preferentially over loop diuretics, which increase it [59]. If hypoparathyroidism is present, recombinant human parathyroid hormone (rhPTH, Natpara), commercially available in the United States, may be indicated.

#### Po_4_^−3^

Due to the readiness with which it combines with oxygen, phosphorus (P) exists in the body as phosphate (PO_4_) [60], where it constitutes the chief intracellular anion. However, the bulk of total body phosphate (85%), which is partially accessible through bone resorption, is found in the skeleton [61]. Normal phosphate concentrations are between 2.5 and 4.5 mg/dL.

Potential mechanisms of hypophosphatemia (<2.5 mg/dL) in platinum-treated patients are low magnesium, increased renal excretion due to tubular dysfunction, decreased intestinal absorption from diarrhea, tumor growth (presumably due to consumption of phosphate by the tumor cells), nutrient deprivation from cachexia, hyperparathyroidism (which causes phosphate levels to fall and calcium to rise), hypercalcemia of malignancy, alcoholism due to generalized proximal tubule dysfunction [62], administration of glucose and insulin, which stimulates carbohydrate metabolism and with it the transport of phosphate into cells, and several drugs including diuretics, glucocorticoids, aminoglycosides, antiretrovirals and aluminum-containing antacids, which chelate phosphates. Rare mesenchymal tumors also cause hypophosphatemia due to paraneoplastic secretion of the phosphaturic hormone, fibroblast growth factor 23 [63], referred to as tumor-induced osteomalacia (TIO).

**Fig. 5:**
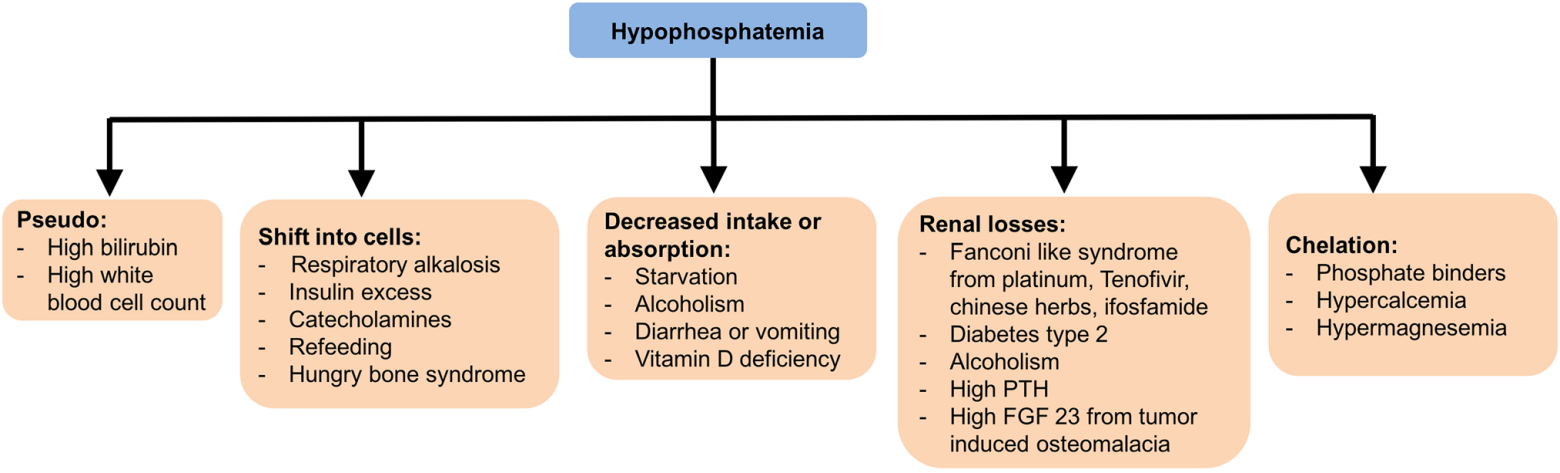
Hypophosphatemia causes briefly illustrated

A partial list of causes, which may precipitate or exacerbate hypophosphatemia, is shown in Table 4 and Fig. 5.

**Table 4:** Hypophosphatemia Causes

Decreased entry	Loss	Cellular shift	Paraneoplastic
Hyperparathyroidism	Renal tubular dysfunction (e.g., drugs, alcoholism)	Diabetic ketoacidosis	TIO
Magnesium deficiency	Chelation	Administration of glucose and insulin	
	Aluminum-containing antacids		
	Hypercalcemia		
Vitamin D deficiency	Loop and thiazide diuretics	Respiratory alkalosis	
Cachexia	Vomiting and diarrhea	Refeeding after prolonged undernutrition	
Cushing’s syndrome due to increased glucocorticoids

### Manifestations

Since ATP is comprised of three high-energy phosphate groups, impaired ATP and energy generation, leading to muscle weakness, is a consequence of hypophosphatemia. Other signs and symptoms, which typically only appear when the deficiency is severe, include bone pain and loss of bone density, hemolysis, cardiac and respiratory failure and rhabdomyolysis.

### Management

**Fig. 6:**
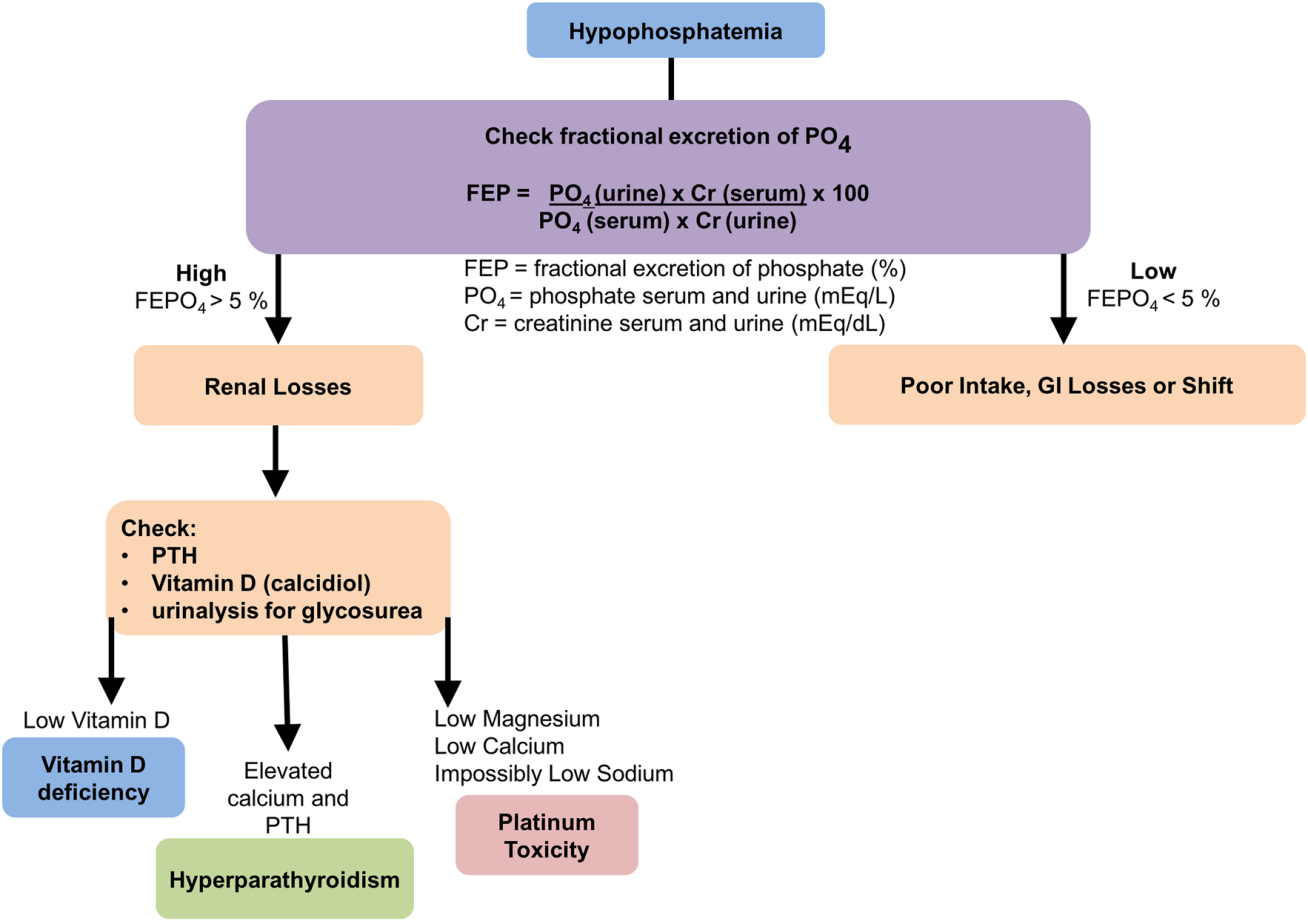
Hypophosphatemia diagnostic algorithm

The general recommendation is to correct severe hypophosphatemia (phosphate levels <0.32 mmol/L) in symptomatic patients with the proviso that iv repletion may induce a life-threatening hypokalemia [64]. Moderate hypophosphatemia is treatable with oral supplementation of phosphate bearing in mind that active vitamin D is required for intestinal absorption. Typical oral supplementation amounts are three times the normal daily intake, with advised amounts of 2.5–3.5 g (80–110 mmol) per day, divided over two to three doses. (Fig. 6).

## Hyponatremia

### Background

Hyponatremia, a serious electrolyte disorder associated with life-threatening neurological complications, is defined as a decrease in serum concentration of sodium, the major extracellular cation <136 mEq/L due to an excess of water relative to solute [65]. Serum sodium (Na^+^) concentration is tightly regulated principally by the antidiuretic actions of arginine vasopressin (AVP), which binds to the V2 receptor in the renal collecting ducts [66] and stimulates translocation of the aquaporin-2 water channels, resulting in increased free-water reabsorption [67]. The mineralocorticoid hormone, aldosterone, also regulates sodium (and potassium) levels via the amiloride‐sensitive sodium channel and Na–K ATPase pump [68].

Platinum chemotherapy has been associated with both renal salt-wasting syndrome (RSWS) and inappropriate antidiuretic hormone secretion (SIADH [69]). Since the treatment for RSWS is supplement of salt, while SIADH requires fluid restriction, it is important to make the distinction. The incidence of platinum-induced hyponatremia may be as high as 43% [70].

### Mechanism

The mechanism by which platinum chemotherapy leads to renal salt wasting is not well understood. Platinum-induced nephrotoxicity is related to accumulation [71, 72] in the proximal and distal epithelial cells (however, it preferentially targets the proximal cells [73]) with subsequent inflammation, functional alteration of cell membrane transporters and apoptosis. Therefore, the most likely site for depressed renal sodium absorption in RSWS is the proximal nephron where the bulk of filtered Na is reabsorbed. Decreased proximal reabsorption results in the delivery of greater amounts of sodium to the distal nephron; however, with platinum-mediated damage of the distal tubule [74], the expected compensatory distal increase in sodium transport is impaired or prevented, leading to net Na excretion.

In addition to direct renal tubular toxicity, platinum agents may also cause SIADH; however, several tumors including lung, breast and head and neck traditionally treated with platinum doublets are also associated with SIADH [75], which complicates the clinical picture.

### Manifestations and management

The main clinical manifestations of renal salt-wasting syndrome are hyponatremia, increased urinary sodium, increased urine output and hypovolemia, while SIADH is characterized by hyponatremia, increased urinary sodium, normal urine output and euvolemia. Orthostatic hypotension or tachycardia, dry mucus membranes and poor skin turgor [76] as well as a high blood urea nitrogen (BUN) suggest volume depletion (hypovolemia), while a low BUN, normal skin turgor, moist mucus membranes and absence of orthostatic hypotension or tachycardia are supportive of euvolemia [77]. One potential clue to differentiate cancer-related SIADH from platinum-mediated RSWS is that the latter is more likely to be accompanied by other electrolyte abnormalities such as hypomagnesemia, hypokalemia, hypophosphatemia and hypocalcemia [70].

The distinction between the two clinical entities is not academic because the treatment of choice for RSWS is hypertonic saline supplementation, while for SIADH it is fluid restriction, which is detrimental and possibly even lethal for a patient with RSWS. As an alternative to fluid restriction, since adequate hydration is necessary to prevent nephrotoxicity in cisplatin-treated patients, a vaptan (antidiuretic hormone receptor antagonist, e.g., tolvaptan) may be trialed [78] (Table 5).

**Table 5:** Summary RSWS vs. SIADH

	RSWS	SIADH
Volume status	Hypovolemic	Normovolemic (or possibly hypervolemic)
Serum sodium Concentration	Decreased	Decreased
Urine sodium Concentration	Increased	Increased
Urine output	Increased	Normal
Mechanism	Excess secretion of sodium and water due to tubular necrosis	Water retention due to elevated ADH (vasopressin) secretion from tumor

The mostly neurological symptoms associated with hyponatremia (e.g., headache, nausea, vomiting, lethargy, disorientation) are related to the fall in serum tonicity, which favors water movement into the brain, potentially resulting in cerebral edema. However, because the brain’s adaptive reaction to counteract the edema is extrusion of inorganic and organic solutes, a too rapid correction of the serum sodium (>0.5 mmol/L/h), especially when the hyponatremia has been present for >48 h, may result in a pathology called osmotic demyelination syndrome, which is characterized by motor abnormalities, mental state disturbances and coma [76, 79].

## Conclusion

Platinum agents are a cornerstone of treatment for approximately half of all cancer patients. Their widespread use requires that clinicians of all stripes, not just oncologists, should be aware of their side effects, especially as primary providers increasingly figure into the care of cancer patients. Besides the well-publicized myelosuppressive toxicities, platinum agents are also commonly associated with specific electrolyte deficiencies, in particular magnesium, that in turn directly or indirectly interferes with absorption and/or promotes excretion of calcium, potassium and phosphates as well as global electrolyte deficiencies secondary to a Fanconi-like tubulopathy. Fanconi’s syndrome is a genetic disease of the proximal renal tubules associated with metabolic acidosis, hypokalemia, hypophosphatemia, glucosuria and proteinuria [80]. These electrolyte imbalances may be associated with significant but preventable morbidity and mortality; hence, their prompt recognition and correction is essential to good patient outcomes and provides the *raison d’être * for this review.

Since magnesium deficiency is so common with platinum administration and since other comorbidities (vomiting, diarrhea, diabetes mellitus, coronary artery disease) or concomitant medications (diuretics, proton pump inhibitors, stool softeners, etc.) frequently encountered in the care of cancer patients exacerbates the risk, it is reasonable to consider whether the preventive prescription of routine oral and IV magnesium supplementation, absent a contraindication, is indicated with all platinum agents, given the cardiac and renal consequences of low Mg^+2^.

However, with the exception of magnesium, preventative supplementation is not indicated with calcium, potassium, phosphorus and sodium, given the potential to increase the risk of other toxicities (e.g., cardiac arrhythmias with calcium administration). In these cases, prevention consists of early diagnosis and treatment.

However, the takeaway message from this review is that the iatrogenic residua from electrolyte deficiencies during platinum therapy, which include weakness, fatigue, malaise, cardiac arrhythmias and tetany, are not easily diagnosed at an early or late stage without specific foreknowledge (and in some cases even with it), since the clinical signs and symptoms, which are protean and overlap with myriad conditions, may be written off as “complications” of the cancer; for this reason, a higher level of suspicion is warranted and physicians should be on the lookout for early indications of these deficiencies, especially in patients with underlying illnesses or concomitant medications that may exacerbate them, before they worsen and potentially result in worse morbidity and even mortality, if left untreated.

Therefore, on the premise that forewarned is forearmed and given that the goal of cancer therapy is to maximize tumor control while minimizing systemic toxicity, the purpose of this review is to help clinicians, especially “generalists” pressed into service as follow-up cancer care providers, ameliorate or prevent these treatable sequelae of platinum-based treatment, which will ostensibly contribute to better survival, fewer hospitalizations, better patient experience and, ultimately, improved quality of life.
